# Information-Theoretic Generalization Bounds for Batch Reinforcement Learning

**DOI:** 10.3390/e26110995

**Published:** 2024-11-18

**Authors:** Xingtu Liu

**Affiliations:** School of Computing Science, Simon Fraser University, 8888 University Dr W, Burnaby, BC V5A 1S6, Canada; xingtu_liu@sfu.ca

**Keywords:** reinforcement learning, learning theory, generalization, mutual information

## Abstract

We analyze the generalization properties of batch reinforcement learning (batch RL) with value function approximation from an information-theoretic perspective. We derive generalization bounds for batch RL using (conditional) mutual information. In addition, we demonstrate how to establish a connection between certain structural assumptions on the value function space and conditional mutual information. As a by-product, we derive a *high-probability* generalization bound via conditional mutual information, which was left open and may be of independent interest.

## 1. Introduction

Generalization is a fundamental concept in statistical machine learning. It measures how well a learning system performs on unseen data after being trained on a finite dataset. Effective generalization ensures that the learning approach captures the essential patterns in the data. Generalization in supervised learning has been studied for several decades. However, in reinforcement learning (RL), agnostic learning is generally infeasible and realizability is not a sufficient condition for efficient learning. Consequently, the study of generalization in RL poses more challenges.

In this work, we focus on batch reinforcement learning (batch RL), a branch of reinforcement learning where the agent learns a policy from a fixed dataset of previously collected experiences. This setting is favorable when online interaction is expensive, dangerous, or impractical. Batch RL, despite being a special case of supervised learning, still presents distinct challenges due to the complex temporal structures inherent in the data.

Originating from the work of [1,2], an information-theoretic framework has been developed to bound the generalization error of learning algorithms using the mutual information between the input dataset and the output hypothesis. This methodology formalizes the intuition that overfitted learning algorithms are less likely to generalize effectively. Unlike traditional approaches such as VC-dimension and Rademacher complexity, this information-theoretic framework offers the significant advantage of capturing all dependencies on the data distribution, hypothesis space, and learning algorithm. Given that reinforcement learning is a learning paradigm in which all the aforementioned aspects differ significantly from those in supervised learning, we believe this novel approach will provide us with more profound insights.

## 2. Preliminaries

### 2.1. Batch Reinforcement Learning with Function Approximation

**Figure 1:**
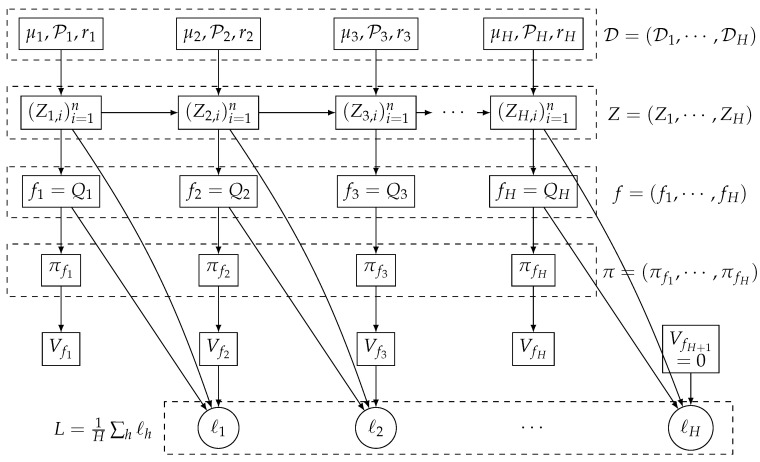
Directed graph representing the training process in Batch RL under episodic MDP.

An episodic Markov decision process (MDP) is defined by M(S,A,P,r,H). We use Δ(X) to denote the set of the probability distribution over the set X. M(S,A,P,r,H) is specified by a finite state space S, a finite action space A, transition functions $P_{h}:S\times A\to \Delta \left(S\right)$ at step h∈[H], reward function $r_{h}:S\times A\to R$ at step *h*, and *H* is the number of steps in each episode. We assume the reward is bounded, i.e., $r_{h}\left(s,a\right)\in \left[0,1\right]$ (For rewards in $\left[R_{\min},R_{\max}\right]$ simply rescales these bounds.), ∀(s,a,h). See Figure 1 for a graphical illustration.

Let $\pi ={\left\{\pi_{h}:S\to \Delta \left(A\right)\right\}}_{h\in [H]}$, where $\pi_{h}\left(\cdot |s\right)$ is the action distribution for policy π at state *s* and step *h*. Given a policy π, the value function $V_{h}^{\pi }:S\to R$ at step *h* is defined as

$$V_{h}^{\pi }\left(s\right):=E_{\pi }\left[\sum_{h^{\prime }=h}^{H}r_{h^{\prime }}\left(s_{h^{\prime }},a_{h^{\prime }}\right)|s_{h}=s\right].$$

The action-value function $Q_{h}^{\pi }:S\times A\to R$ at step *h* is defined as

$$Q_{h}^{\pi }\left(s,a\right):=E_{\pi }\left[\sum_{h^{\prime }=h}^{H}r_{h^{\prime }}\left(s_{h^{\prime }},a_{h^{\prime }}\right)|s_{h}=s,a_{h}=a\right].$$

The Bellman operators $T_{h}^{\pi }$ and $T_{h}^{*}$ project functions forward by one step through the following dynamics:

$$\left(T_{h}^{\pi }\right)\left(s,a\right)=r_{h}\left(s,a\right)+E_{s^{\prime }∼P_{h}\left(\cdot |s,a\right)}\left[E_{a^{\prime }∼\pi \left(\cdot |s^{\prime }\right)}\left[Q\left(s^{\prime },a^{\prime }\right)\right]\right],$$

$$\left(T_{h}^{*}\right)\left(s,a\right)=r_{h}\left(s,a\right)+E_{s^{\prime }∼P_{h}\left(\cdot |s,a\right)}\left[\max_{a^{\prime }}Q\left(s^{\prime },a^{\prime }\right)\right].$$

Now, we denote the dataset $Z=\{\left(s,a,r,s^{\prime },h\right)\}$, where $\left(s,a\right)∼\mu_{h}$, $r∼r_{h}\left(s,a\right)$, and $s^{\prime }∼P_{h}\left(\cdot |s,a\right)$ for a fixed *h*. We also denote $D=D_{1}\times \cdots \times D_{H}$, where $\left(s,a,r,s^{\prime },h\right)∼D_{h}$. We consider batch RL with value function approximation. The learner is given a function class $F=F_{1}\times \cdots \times F_{H}$ to approximate the optimal *Q*-value function. Denote $f=(f_{1},\cdots ,f_{H})\in F$. As no reward is collected in the (H+1)th step, we set $f_{H+1}=0$. For each f∈F, define $\pi_{f}={\left\{\pi_{f_{h}}\right\}}_{h=1}^{H}$, where $\pi_{f_{h}}\left(a|s\right)=1\left[a=\underset{a^{\prime }}{\arg\max}f_{h}\left(s,a^{\prime }\right)\right]$. Next, we introduce the Bellman error and its empirical version.

**Definition** **1** (Bellman error)**.** *Under data distribution μ, we define the Bellman error of function $f=(f_{1},\cdots ,f_{H})$ as*

(1) $$E\left(f\right):=\frac{1}{H}\sum_{h=1}^{H}{∥f_{h}-T_{h}^{★}f_{h+1}∥}_{\mu_{h}}^{2}.$$

**Definition** **2** (Mean squared empirical Bellman error (MSBE))**.** *Given a dataset Z∼D, we define the Mean squared empirical Bellman error (MSBE) of function $f=(f_{1},\cdots ,f_{H})$ as*

$$\begin{array}{c} L\left(f,Z\right)=\frac{1}{H}\sum_{h=1}^{H}\frac{1}{n}\sum_{\left(s,a,r,s^{\prime },h\right)\in Z_{h}}{\left(f_{h}\left(s,a\right)-r-V_{f_{h+1}}\left(s^{\prime }\right)\right)}^{2} \end{array}$$

*where* $V_{f_{h+1}}\left(s\right):=\max_{a\in A}f_{h+1}\left(s,a\right)$.

For convenience, we denote $\ell \left(f_{h},Z_{h}\right)=\frac{1}{n}\sum_{\left(s,a,r,s^{\prime },h\right)\in Z_{h}}{\left(f_{h}\left(s,a\right)-r-V_{f_{h+1}}\left(s^{\prime }\right)\right)}^{2}$.

Bellman error is used in RL as a surrogate loss function to minimize the difference between the estimated value function and the true value function under a policy. The Bellman error serves as a proxy for the optimality gap, which is the difference between the current value function and the optimal value function. Under the concentrability assumption, minimizing the Bellman error is able to reduce the optimality gap.

**Lemma** **1** (Bellman error to value suboptimality [3])**.** *If there exists a constant C, such that for any policy π*

$$\underset{\left(s,a,h)\in S\times A\times [H\right]}{\sup}\frac{dP_{h}^{\pi }}{d\mu_{h}}\left(s,a\right)\leq C$$

*then for any f∈F, we have*

$$V_{1}^{*}\left(s_{1}\right)-V_{1}^{\pi_{f}}\left(s_{1}\right)\leq 2H\sqrt{C\cdot E(f)}.$$

We note that L(f,Z) is a biased estimate of E(f). A common solution is to use the double sampling method, where for each state and action in the sample, at least two next states are generated [3,4,5], and define the unbiased MSBE as:

$$L_{\mathrm{DS}}\left(f,\tilde{Z}\right)=\frac{1}{nH}\sum_{\left(s,a,r,s^{\prime },{\tilde{s}}^{\prime },h\right)\in \tilde{Z}}\left[{\left(f_{h}\left(s,a\right)-r-V_{f_{h+1}}\left(s^{\prime }\right)\right)}^{2}-\frac{1}{2}{\left(V_{f_{h+1}}\left(s^{\prime }\right)-V_{f_{h+1}}\left({\tilde{s}}^{\prime }\right)\right)}^{2}\right].$$

Note that $L\left(f,Z\right)\in \left[0,4H^{2}\right]$, $L_{\mathrm{DS}}\left(f,\tilde{Z}\right)\in \left[-2H^{2},4H^{2}\right]$, and double sampling does not increase the sample size, except that it requires an additional generated ${\tilde{s}}^{\prime }∼P_{h}\left(\cdot |s,a\right)$. Therefore, the results presented in this paper can be easily extended to the double sampling setting.

### 2.2. Generalization Bounds

**Definition** **3** (Expected generalization bounds)**.** *Given a dataset Z∼D and an algorithm A, let L(A(Z),Z) denote the training loss and let L(A(Z),D) denote the true loss. The expected generalization error is defined as*

$$\left|E_{Z∼D}\left[L\left(A\left(Z\right),Z\right)-L\left(A\left(Z\right),D\right)\right]\right|.$$

**Definition** **4** (High-probability generalization bounds)**.** *Given a dataset Z∼D, and an algorithm A, let L(A(Z),Z) denote the training loss and let L(A(Z),D) denote the true loss. Given a failure probability δ and an error tolerance η, the high-probability generalization error is defined as*

P(|L(A(Z),Z)−L(A(Z),D)|≥η)≤δ.

### 2.3. Mutual Information

First, we define the KL-divergence of two distributions.

**Definition** **5** (KL-Divergence [6])**.** *Let P,Q be two distributions over the space *Ω* and suppose P is absolutely continuous with respect to Q. The Kullback–Leibler (KL) divergence from Q to P is*

$$D\left(P∥Q\right)=E_{X∼P_{X}}\left[\log\frac{P_{X}}{Q_{X}}\right],$$

*where $P_{X}$ and $Q_{X}$ denote the probability mass/density functions of P and Q on X, respectively.*

Based on KL-divergence, we can define mutual information and conditional mutual information as follows.

**Definition** **6** ([6])**.** *Let X, Y, and Z be arbitrary random variables, and let $D_{\mathrm{KL}}$ denote the Kullback–Leibler (KL) divergence. The mutual information between X and Y is defined as:*

$$I\left(X;Y\right):=D_{\mathrm{KL}}\left(P_{X,Y}∥P_{X}⊗P_{Y}\right).$$

*The conditional mutual information is defined as:*

$$I\left(X;Y|Z\right):=E_{Z}\left[D_{\mathrm{KL}}\left(P_{X,Y|Z}∥P_{X|Z}⊗P_{Y|Z}\right)\right].$$

Next, we introduce Rényi’s α-Divergence, which is a generalization of KL-divergence. Rényi’s α-Divergence has found many applications, such as hypothesis testing, differential privacy, several statistical inference, and coding problems [7,8,9,10].

**Definition** **7** (Rényi’s α-Divergence [11])**.** *Let (Ω,F,P), (Ω,F,Q) be two probability spaces. Let α>0 be a positive real different from 1. Consider a measure μ, such that P≪μ and Q≪μ (such a measure always exists, e.g., μ=(P+Q)/2) and denote with p,q the densities of P,Q with respect to μ. The α–divergence of P from Q is defined as follows:*

$$D_{\alpha }\left(P∥Q\right)=\frac{1}{\alpha -1}\log\int p^{\alpha }q^{1-\alpha }\;d\mu .$$

Note that the above definition is independent of the chosen measure μ. With the definition of Rényi’s α-divergence, we are ready to state the definitions of α-mutual information and α-conditional mutual information.

**Definition** **8** (α-mutual information [7])**.** *Let X,Y be two random variables jointly distributed according to $P_{XY}$. Let $Q_{Y}$ be any probability measure over Y. For α>0, the α-mutual information between X and Y is defined as follows:*

$$I_{\alpha }\left(X;Y\right)=\min_{Q_{Y}}D_{\alpha }\left(P_{XY}∥P_{X}⊗Q_{Y}\right).$$

**Definition** **9** (Conditional α-mutual information)**.** *Let X,Y,Z be three random variables jointly distributed according to $P_{XYZ}$. Let $Q_{Y|Z}$ be any probability measure over Y|Z. For α>0, a conditional α-mutual information of order α between X and Y given Z is defined as follows:*

$$I_{\alpha }^{Y|Z}\left(X;Y|Z\right)=\min_{Q_{Y|Z}}D_{\alpha }\left(P_{XYZ}∥P_{X|Z}⊗Q_{Y|Z}⊗P_{Z}\right).$$

## 3. Generalization Bounds via Mutual Information

Mutual information bounds provide a direct link between the generalization error and the amount of information shared between the training data and the learned hypothesis. This offers a clear information-theoretic understanding of how overfitting can be controlled by reducing the dependency on the training data. Mutual information bounds are applicable to a wide range of learning algorithms and settings, including those with unbounded loss functions and complex hypothesis spaces. Moreover, the use of mutual information can simplify the analysis of generalization compared with traditional methods, particularly in cases where those traditional measures are difficult to compute. See Appendix A for related work.

**Theorem** **1** ([2])**.** *Let D be a distribution on Z. Let A:Z→W be a randomized algorithm. Let ℓ:W×Z→R be a loss function, which is σ-subgaussian with respect to Z. Let L:W×Z→R be the empirical risk. Then*

$$\left|E_{Z∼D}\left[L\left(A\left(Z\right),Z\right)-L\left(A\left(Z\right),D\right)\right]\right|\leq \sqrt{\frac{2\sigma^{2}}{n}I\left(A\left(Z\right);Z\right)}.$$

The above theorem provides a bound on the expected generalization error. High-probability generalization bounds can be obtained using the α-mutual information. Note that the α-mutual information shares many properties with standard mutual information.

**Proposition** **1** ([7])**.** *For discrete random variables X and Y, the following holds:*

*(i)*  *Data Processing Inequality: given α>0, $I_{\alpha }\left(X,Z\right)\leq \min\left\{I_{\alpha }\left(X,Y\right),I_{\alpha }\left(Y,Z\right)\right\}$ if the Markov chain X−Y−Z holds.*
*(ii)* 
$I_{\alpha }\left(X;Y\right)\;is\;\text{non-decreasing}\;in\;\alpha .$

*(iii)* 
$I_{\alpha }\left(X,Y\right)\leq \min\left\{\log|X|,\log|Y|\right\}.$

*(iv)*  *$I_{\alpha }\left(X,Y\right)\geq 0$ with equality iff X and Y are independent.*

**Theorem** **2** ([11])**.** *Let D be a distribution on Z. Let A:Z→W be a randomized algorithm. Let ℓ:W×Z→R be a loss function which is σ-subgaussian with respect to Z. Let L:W×Z→R be the empirical risk. Given η,δ∈(0,1) and fix α≥1, if the number of samples n satisfies*

$$n\geq \frac{2\sigma^{2}}{\eta^{2}}\left(I_{\alpha }\left(A\left(Z\right),Z\right)+\log2+\frac{\alpha }{\alpha -1}\log\left(\frac{1}{\delta }\right)\right).$$

*then, we have*

$$P\left(|L(A(Z),Z)-L(A(Z),D)|\leq \eta \right)\geq 1-\delta .$$

The mutual information bound can be infinite in some cases and thus be vacuous. To address this, the conditional mutual information (CMI) approach was introduced. CMI bounds normalize the information content for each data point, preventing the problem of infinite information content, particularly in continuous data distributions. This makes CMI a more robust and applicable method in scenarios where mutual information would otherwise be unbounded.

**Definition** **10.** *Let $Z∼D^{2n}$ consist of 2n samples drawn independently from D. Let $U\in {\left\{0,1\right\}}^{n}$ be uniformly random and independent from Z and the randomness of A. Define $Z_{U}\in Z$, such that ${\left(Z_{U}\right)}_{i}$ is the ${\left(2i-U_{i}\right)}^{\mathrm{th}}$ sample in Z—that is, $Z_{U}$ is the subset of Z indexed by U. The conditional mutual information of A with respect to D is defined as $I(A\left(Z_{U}\right);U|Z).$*

**Theorem** **3** ([12])**.** *Let D be a distribution on Z. Let A:Z→W be a randomized algorithm. Let L:W×Z→R be a function, such that $|L\left(w,z_{1}\right)-L\left(w,z_{2}\right)|\leq \Delta \left(z_{1},z_{2}\right)$ for all $z_{1},z_{2}\in Z$ and w∈W given $\Delta :Z^{2}\to R$. Let $U\in {\left\{0,1\right\}}^{n}$ be uniformly random. Then*

$$\left|E_{Z∼D}\left[L\left(A\left(Z_{U}\right),Z_{U}\right)-L\left(A\left(Z_{U}\right),D\right)\right]\right|\leq \sqrt{\frac{2E_{z_{1},z_{2}}\left[\Delta {\left(z_{1},z_{2}\right)}^{2}\right]}{n}I\left(A\left(Z_{U}\right);U|Z\right)}.$$

Another advantage of the CMI bounds is that they can be derived from various concepts such as VC-dimension, compression schemes, stability, and differential privacy, offering a unified framework for generalization analysis. However, because CMI is defined as an expectation, i.e., $I\left(X;Y|Z\right):=E_{Z}\left[D_{\mathrm{KL}}\left(P_{X,Y|Z}∥P_{X|Z}⊗P_{Y|Z}\right)\right]$, the above theorem does not provide a high-probability bound. Modifying this framework to ensure high-probability guarantees was left as future work in [12]. In the following, we use conditional α-mutual information to address this issue.

**Theorem** **4.** *Let $U\in {\left\{0,1\right\}}^{n}$ be uniformly random. Given a dataset $Z∼D^{2n}$ consists of 2nH samples. Let $A:Z_{U}\to W$ be a randomized algorithm. Let ℓ:W×Z→R be a loss function which is σ-subgaussian with respect to Z. Let $L:W\times Z_{U}\to R$ be the empirical risk. Given η,δ∈(0,1) and fix α≥1, if the number of samples n satisfies*

$$n\geq \frac{2\sigma^{2}}{\eta^{2}}\left(I_{\alpha }^{A(Z_{U})|Z}\left(A\left(Z_{U}\right);U|Z\right)+\log2+\frac{\alpha }{\alpha -1}\log\left(\frac{1}{\delta }\right)\right)$$

*then, we have*

$$P\left(|L\left(A\left(Z_{U}\right),Z_{U}\right)-L\left(A\left(Z_{U}\right),D\right)|\leq \eta \right)\geq 1-\delta .$$

**Proof.**  Let $\left(X\times Y\times Z,F,P_{XYZ}\right)$ be a probability space, and let Q(X|Z) be the set of conditional probability measures $Q_{X|Z}$, such that $P_{XYZ}\ll P_{Z}Q_{X|Z}P_{Y|Z}$. Given E∈F and z∈Z,x∈X, let $E_{z,x}=\left\{y\in Y:\left(x,y,z\right)\in E\right\}$. We first prove that for a fixed α≥1,

(2) $$\begin{array}{c} P_{XYZ}\left(E\right)\leq E_{Z}{\left[{\mathrm{ess}\;\sup}_{Q_{X|Z}\in Q\left(X|Z\right)}P_{Y|Z}\left(E_{Z,X}\right)\right]}^{\frac{\alpha -1}{\alpha }}\exp\left(\frac{\alpha -1}{\alpha }I_{\alpha }^{X|Z}\left(X;Y|Z\right)\right). \end{array}$$

Using the Radon–Nikodym derivative of $P_{XYZ}$ with respect to the product measure $P_{Z}Q_{X|Z}P_{Y|Z}$, we have

$$\begin{array}{cc} P_{XYZ}\left(E\right) & =E_{P_{Z}Q_{X|Z}P_{Y|Z}}\left[\frac{dP_{XYZ}}{dP_{Z}Q_{X|Z}P_{Y|Z}}I_{E}\right] \end{array}$$

where $I_{E}$ is the indicator function of the event *E*. Next, we introduce three sets of exponents $\alpha^{\prime \prime },\alpha^{\prime },\alpha$, and $\gamma^{\prime \prime },\gamma^{\prime },\gamma$, such that

$$\frac{1}{\alpha^{\prime \prime }}+\frac{1}{\gamma^{\prime \prime }}=\frac{1}{\alpha^{\prime }}+\frac{1}{\gamma^{\prime }}=\frac{1}{\alpha }+\frac{1}{\gamma }=1.$$

By applying Hölder’s inequality three times to separate the different components of the expectation, we derive

$$\begin{array}{cc} \; & E_{P_{Z}Q_{X|Z}P_{Y|Z}}\left[\frac{dP_{XYZ}}{dP_{Z}Q_{X|Z}P_{Y|Z}}I_{E}\right]\\ \; & \leq E_{P_{Z}}^{\frac{1}{\alpha^{\prime \prime }}}\left[E_{Q_{X|Z}}^{\frac{\alpha^{\prime \prime }}{\alpha^{\prime }}}\left[E_{Q_{Y|Z}}^{\frac{\alpha }{\alpha^{\prime }}}\left[{\left(\frac{dP_{XYZ}}{dP_{Z}Q_{X|Z}P_{Y|Z}}\right)}^{\alpha }\right]\right]\right]E_{P_{Z}}^{\frac{1}{\gamma^{\prime \prime }}}\left[E_{Q_{X|Z}}^{\frac{\gamma^{\prime \prime }}{\gamma^{\prime }}}\left[E_{P_{Y|Z}}^{\frac{\gamma^{\prime }}{\gamma }}\left[I_{E}^{\gamma }\right]\right]\right]. \end{array}$$

By setting $\alpha^{\prime \prime }=\alpha$ and $\alpha^{\prime }=1$,

$$\begin{array}{c} E_{P_{Z}}^{\frac{1}{\alpha^{\prime \prime }}}\left[E_{Q_{X|Z}}^{\frac{\alpha^{\prime \prime }}{\alpha^{\prime }}}\left[E_{Q_{Y|Z}}^{\frac{\alpha }{\alpha^{\prime }}}\left[{\left(\frac{dP_{XYZ}}{dP_{Z}Q_{X|Z}P_{Y|Z}}\right)}^{\alpha }\right]\right]\right]\leq \exp\left(\frac{\alpha -1}{\alpha }I_{\alpha }^{X|Z}\left(X;Y|Z\right)\right). \end{array}$$

Since $\alpha^{\prime }=1$ and $\frac{1}{\alpha^{\prime }}+\frac{1}{\gamma^{\prime }}=1$, we have $\gamma^{\prime }\to \infty$. As $\gamma^{\prime }\to \infty$, $E_{Q_{X|Z}}^{\frac{\gamma^{\prime \prime }}{\gamma^{\prime }}}\left[E_{P_{Y|Z}}^{\frac{\gamma^{\prime }}{\gamma }}\left[I_{E}^{\gamma }\right]\right]$ tends to the essential supremum

$$\begin{array}{c} {\mathrm{ess}\;\sup}_{Q_{X|Z}\in Q\left(X|Z\right)}P_{Y|Z}\left(E_{Z,X}\right). \end{array}$$

As $\frac{1}{\gamma^{\prime \prime }}=\frac{\alpha -1}{\alpha }$, we have

$$\begin{array}{c} E_{P_{Z}}^{\frac{1}{\gamma^{\prime \prime }}}\left[E_{Q_{X|Z}}^{\frac{\gamma^{\prime \prime }}{\gamma^{\prime }}}\left[E_{P_{Y|Z}}^{\frac{\gamma^{\prime }}{\gamma }}\left[I_{E}^{\gamma }\right]\right]\right]\leq E_{Z}{\left[{\mathrm{ess}\;\sup}_{Q_{X|Z}\in Q\left(X|Z\right)}P_{Y|Z}\left(E_{Z,X}\right)\right]}^{\frac{\alpha -1}{\alpha }}. \end{array}$$

Thus, Equation (2) holds by combining all of the inequalities.

Now, let $X=A(Z_{U})$ and Y=U. Consider the event

$$E=\left\{\left(X,Y,Z\right):\left|L\left(X,Z_{Y}\right)-E_{Y}\left[L\left(X,D\right)\right]\right|\geq \eta \right\},$$

where $L(X,Z_{Y})$ denotes the empirical risk defined as the average of *n* loss functions, and each loss function is σ-subgaussian. We can express $E_{Z,X}$, the fibers of *E*, with respect to *Z* and *X*, as

$$E_{Z,X}=\left\{Y:\left|L\left(X,Z_{Y}\right)-E_{Y}\left[L\left(X,D\right)\right]\right|\geq \eta \right\}.$$

For any fixed *Z* and *X*, the random variable *Y* remains independent of *Z* and *X* under any $Q_{X|Z}\in Q\left(X|Z\right)$. Now, using Hoeffding’s inequality, for every *X* and *Z*,

(3) $$\begin{array}{c} P_{Y}\left(E_{Z,X}\right)\leq 2\exp\left(\frac{-n\eta^{2}}{2\sigma^{2}}\right). \end{array}$$

Therefore, from Equations (2) and (3),

$$\begin{array}{cc} P(E) & \leq 2\exp\left(\frac{\alpha -1}{\alpha }\cdot \frac{-n\eta^{2}}{2\sigma^{2}}\right)\exp\left(\frac{\alpha -1}{\alpha }I_{\alpha }^{A(Z_{U})|Z}\left(A\left(Z_{U}\right);U|Z\right)\right)\\ \; & =2\exp\left(\frac{\alpha -1}{\alpha }\left(I_{\alpha }^{A(Z_{U})|Z}\left(A\left(Z_{U}\right);U|Z\right)-\frac{-n\eta^{2}}{2\sigma^{2}}\right)\right). \end{array}$$

Lastly, by setting

$$n\geq \frac{2\sigma^{2}}{\eta^{2}}\left(I_{\alpha }^{A(Z_{U})|Z}\left(A\left(Z_{U}\right);U|Z\right)+\log2+\frac{\alpha }{\alpha -1}\log\left(\frac{1}{\delta }\right)\right)$$

we obtain the desired conclusion. □

## 4. Information-Theoretic Generalization Bounds for Batch RL

We now provide expected and high-probability generalization bounds for batch RL. The generalization bounds are derived from mutual information between the training data and the learned hypothesis. As mutual information bounds consider the data, algorithm, and hypothesis space comprehensively, they support the design of efficient learning algorithms and fine-grained theoretical analysis.

**Theorem** **5.** *Given that dataset $Z∼D^{n}$ consists of nH samples, for any batch RL algorithm A with output $A\left(Z\right)=f=\left(f_{1},\cdots ,f_{H}\right)\in F$, the expected generalization error for the mean squared empirical Bellman error (MSBE) loss is upper bounded by*

$$\begin{array}{c} \left|E_{Z∼D}\left[L\left(A\left(Z\right),Z\right)-L\left(A\left(Z\right),D\right)\right]\right|\leq \sqrt{\frac{2H^{2}\sum_{h=1}^{H}I\left(f_{h};Z_{h}\right)}{n}}. \end{array}$$

**Proof.**  We first recall the Donsker—Varadhan variational representation ([13]) of the KL-divergence between any two probability measures π and ρ on a common measurable space (Ω,F)

$$\begin{array}{c} D_{\mathrm{KL}}\left(\pi ∥\rho \right)=\underset{F}{\sup}\left\{\int_{\Omega }F\;d\pi -\log\int_{\Omega }e^{F}\;d\rho \right\} \end{array}$$

where the supremum is over all measurable functions F:Ω→R, such that $e^{F}\in L^{1}\left(\rho \right)$.

Let be $Z=Z_{1}\cup \cdots \cup Z_{H}$ be a dataset where $Z_{h}=\left\{\left(s,a,r,s^{\prime },h\right)\right\}∼D_{h}$. Let $A\left(Z\right)=f=\left(f_{1},\cdots ,f_{H}\right)\in F$ be the output of some batch RL algorithm A. Let ${\tilde{f}}_{h}$ and ${\tilde{Z}}_{h}$ be the independent copies of $f_{h}$ and $Z_{h}$. Let

$$\begin{array}{cc} L(f,Z) & =\frac{1}{H}\sum_{h=1}^{H}\ell \left(f_{h},Z_{h}\right)\\ \; & =\frac{1}{H}\sum_{h=1}^{H}\frac{1}{n}\sum_{\left(s,a,r,s^{\prime },h\right)\in Z_{h}}{\left(f_{h}\left(s,a\right)-r-V_{f_{h+1}}\left(s^{\prime }\right)\right)}^{2}. \end{array}$$

Now, we have

$$\begin{array}{cc} I(f_{h};Z_{h}) & =D_{\mathrm{KL}}\left(P_{f_{h},Z_{h}}∥P_{f_{h}}⊗P_{Z_{h}}\right)\\ \; & =\underset{g}{\sup}\left\{E_{f_{h},Z_{h}}\left[g\left(f_{h},Z_{h}\right)\right]-\log E_{{\tilde{f}}_{h},{\tilde{Z}}_{h}}\left[e^{g({\tilde{f}}_{h},{\tilde{Z}}_{h})}\right]\right\}\\ \; & \;\;\;\;\;\;(\text{Donsker–Varadhan}\;\mathrm{variational}\;\mathrm{representation})\\ \; & \geq \lambda E_{f_{h},Z_{h}}\left[\ell \left(f_{h},Z_{h}\right)\right]-\log E_{{\tilde{f}}_{h},{\tilde{Z}}_{h}}\left[e^{\lambda \ell ({\tilde{f}}_{h},{\tilde{Z}}_{h})}\right].\;\;\;(\forall \lambda \in R) \end{array}$$

As $\ell \left(f_{h},Z_{h}\right)=\frac{1}{n}\sum_{\left(s,a,r,s^{\prime },h\right)\in Z_{h}}{\left(f_{h}\left(s,a\right)-r-V_{f_{h+1}}\left(s^{\prime }\right)\right)}^{2}$ and ${\left(f_{h}\left(s,a\right)-r-V_{f_{h+1}}\left(s^{\prime }\right)\right)}^{2}\in \left[0,4H^{2}\right]$ for any *h*, it follows that

$$\begin{array}{c} \log E_{{\tilde{f}}_{h},{\tilde{Z}}_{h}}\left[e^{\lambda (\ell \left({\tilde{f}}_{h},{\tilde{Z}}_{h}\right)-E_{{\tilde{f}}_{h},{\tilde{Z}}_{h}}\left[\ell \left({\tilde{f}}_{h},{\tilde{Z}}_{h}\right)\right])}\right]\leq \frac{2\lambda^{2}H^{4}}{n}. \end{array}$$

Thus, we obtain

$$\begin{array}{cc} I(f_{h};Z_{h}) & \geq \lambda \left(E_{f_{h},Z_{h}}\left[\ell \left(f_{h},Z_{h}\right)\right]-E_{{\tilde{f}}_{h},{\tilde{Z}}_{h}}\left[\ell \left({\tilde{f}}_{h},{\tilde{Z}}_{h}\right)\right])]\right)-\frac{2\lambda^{2}H^{4}}{n}\\ \Rightarrow \frac{I(f_{h};Z_{h})}{\lambda }+\frac{2\lambda^{2}H^{4}}{n} & \geq E_{f_{h},Z_{h}}\left[\ell \left(f_{h},Z_{h}\right)\right]-E_{{\tilde{f}}_{h},{\tilde{Z}}_{h}}\left[\ell \left({\tilde{f}}_{h},{\tilde{Z}}_{h}\right)\right])]. \end{array}$$

By optimizing the above inequality over λ>0 and λ<0, respectively, we derive

$$\begin{array}{c} -H^{2}\sqrt{\frac{2I(f_{h};Z_{h})}{n}}\leq E_{f_{h},Z_{h}}\left[\ell \left(f_{h},Z_{h}\right)\right]-E_{{\tilde{f}}_{h},{\tilde{Z}}_{h}}\left[\ell \left({\tilde{f}}_{h},{\tilde{Z}}_{h}\right)\right])]\leq H^{2}\sqrt{\frac{2I(f_{h};Z_{h})}{n}}, \end{array}$$

and thus,

(4) $$\begin{array}{c} \left|E_{f_{h},Z_{h}}\left[\ell \left(f_{h},Z_{h}\right)\right]-E_{{\tilde{f}}_{h},{\tilde{Z}}_{h}}\left[\ell \left({\tilde{f}}_{h},{\tilde{Z}}_{h}\right)\right])]\right|\leq H^{2}\sqrt{\frac{2I(f_{h};Z_{h})}{n}}. \end{array}$$

Finally, we observe that

$$\begin{array}{cc} \left|E_{Z∼D}\left[L\left(A\left(Z\right),Z\right)-L\left(A\left(Z\right),D\right)\right]\right| & =\left|E_{Z∼D}\left[\frac{1}{H}\sum_{h=1}^{H}\ell \left(f_{h},Z_{h}\right)-E_{Z∼D}\left[\frac{1}{H}\sum_{h=1}^{H}\ell \left(f_{h},Z_{h}\right)\right]\right]\right|\\ \; & =\left|\frac{1}{H}\sum_{h=1}^{H}E_{Z_{h}∼D_{h}}\left[\ell \left(f_{h},Z_{h}\right)-E_{Z_{h}∼D_{h}}\left[\ell (f_{h},Z_{h})\right]\right]\right|\\ \; & =\frac{1}{H}\sum_{h=1}^{H}\left|E_{f_{h},Z_{h}}\left[\ell \left(f_{h},Z_{h}\right)\right]-E_{{\tilde{f}}_{h},{\tilde{Z}}_{h}}\left[\ell \left({\tilde{f}}_{h},{\tilde{Z}}_{h}\right)\right])]\right|\\ \; & \leq \frac{1}{H}\sum_{h=1}^{H}H^{2}\sqrt{\frac{2I(f_{h};Z_{h})}{n}}\;\;\;\;\;\;(\mathrm{By}\;\mathrm{Equation}\;(4))\\ \; & =\sqrt{\frac{2H^{2}\sum_{h=1}^{H}I\left(f_{h};Z_{h}\right)}{n}}. \end{array}$$

□

The above result suggests that reducing the mutual information between the dataset $Z_{h}$ and the learned function $f_{h}$ at each step *h* can improve the generalization performance. Note that when the input domain is infinite, mutual information can become unbounded. To address this limitation, an approach based on conditional mutual information was introduced [12]. CMI bounds not only address the issue by normalizing the information content of each data point, but also establish connections with various other generalization concepts, as we will discuss in the next section. We now present a generalization bound using conditional mutual information.

**Theorem** **6.** *Let $U\in {\left\{0,1\right\}}^{n}$ be uniformly random. Given that dataset $Z∼D^{2n}$ consists of 2nH samples, for any batch RL algorithm A with output $A\left(Z_{U}\right)=f=\left(f_{1},\cdots ,f_{H}\right)\in F$, the expected generalization error for the mean squared empirical Bellman error (MSBE) loss is upper bounded by*

$$\begin{array}{c} \left|E_{Z∼D}\left[L\left(A\left(Z_{U}\right),Z_{U}\right)-L\left(A\left(Z_{U}\right),D\right)\right]\right|\leq \sqrt{\frac{2H^{2}\sum_{h=1}^{H}I\left(f_{h};U|Z_{h}\right)}{n}}. \end{array}$$

**Proof.**  Let $U\in {\left\{0,1\right\}}^{n}$ be uniformly random. Let $Z=Z_{1}\cup \cdots \cup Z_{H}$ be a dataset where each $Z_{h}=\left\{\left(s,a,r,s^{\prime },h\right)\right\}∼D_{h}$ consists of 2n samples. Define $Z_{U}={\left(Z_{1}\right)}_{U}\cup \cdots \cup {\left(Z_{H}\right)}_{U}$. Let $A\left(Z_{U}\right)=f=\left(f_{1},\cdots ,f_{H}\right)\in F$ be the output of some batch RL algorithm A. Let ${\bar{f}}_{h}=A{\left(Z_{\bar{U}}\right)}_{h}$, ${\tilde{Z}}_{h}={\left(Z_{h}\right)}_{U}$ and ${\bar{Z}}_{h}={\left(Z_{h}\right)}_{\bar{U}}$. Note that $Z_{h}={\tilde{Z}}_{h}\cup {\bar{Z}}_{h}$. We define the disintegrated mutual information

$$I^{Z}\left(X;Y\right):=D_{KL}(P_{X,Y|Z}∥P_{X}P_{Y}\left|Z\right).$$

Note that $I\left(X;Y|Z\right)=E_{Z}\left[I^{Z}\left(X;Y\right)\right]$. The rest of the proof is analogous to Theorem 5. We have

$$\begin{array}{cc} I^{Z_{h}}\left(f_{h};{\tilde{Z}}_{h}|Z_{h}\right) & =D_{\mathrm{KL}}\left(P_{f_{h},{\tilde{Z}}_{h}|Z_{h}}∥P_{f_{h}|Z_{h}}⊗P_{{\tilde{Z}}_{h}|Z_{h}}\right)\\ \; & =\underset{g}{\sup}\left\{E_{f_{h},{\tilde{Z}}_{h}|Z_{h}}\left[g\left(f_{h},{\tilde{Z}}_{h}\right)\right]-\log E_{{\bar{f}}_{h},{\bar{Z}}_{h}|Z_{h}}\left[e^{g({\bar{f}}_{h},{\bar{Z}}_{h})}\right]\right\}\\ \; & \;\;\;\;\;\;(\text{Donsker–Varadhan}\;\mathrm{variational}\;\mathrm{representation})\\ \; & \geq \lambda E_{f_{h},{\tilde{Z}}_{h}|Z_{h}}\left[\ell \left(f_{h},{\tilde{Z}}_{h}\right)\right]-\log E_{{\bar{f}}_{h},{\bar{Z}}_{h}|Z_{h}}\left[e^{\lambda \ell ({\bar{f}}_{h},{\bar{Z}}_{h})}\right].\;\;\;(\forall \lambda \in R) \end{array}$$

As $\ell \left(f_{h},Z_{h}\right)=\frac{1}{n}\sum_{\left(s,a,r,s^{\prime },h\right)\in Z_{h}}{\left(f_{h}\left(s,a\right)-r-V_{f_{h+1}}\left(s^{\prime }\right)\right)}^{2}$ and ${\left(f_{h}\left(s,a\right)-r-V_{f_{h+1}}\left(s^{\prime }\right)\right)}^{2}\in \left[0,4H^{2}\right]$ for any *h*, it follows that

$$\begin{array}{c} \log E_{{\bar{f}}_{h},{\bar{Z}}_{h}|Z_{h}}\left[e^{\lambda (\ell \left({\bar{f}}_{h},{\bar{Z}}_{h}\right)-E_{{\bar{f}}_{h},{\bar{Z}}_{h}|Z_{h}}\left[\ell \left({\bar{f}}_{h},{\bar{Z}}_{h}\right)\right])}\right]\leq \frac{2\lambda^{2}H^{4}}{n}. \end{array}$$

Thus, we obtain

$$\begin{array}{cc} I^{Z_{h}}\left(f_{h};{\tilde{Z}}_{h}|Z_{h}\right) & \geq \lambda \left(E_{f_{h},{\tilde{Z}}_{h}|Z_{h}}\left[\ell \left(f_{h},{\tilde{Z}}_{h}\right)\right]-E_{{\bar{f}}_{h},{\bar{Z}}_{h}|Z_{h}}\left[\ell \left({\bar{f}}_{h},{\bar{Z}}_{h}\right)\right])]\right)-\frac{2\lambda^{2}H^{4}}{n}\\ \Rightarrow \frac{I^{Z_{h}}\left(f_{h};{\tilde{Z}}_{h}|Z_{h}\right)}{\lambda }+\frac{2\lambda^{2}H^{4}}{n} & \geq E_{f_{h},{\tilde{Z}}_{h}|Z_{h}}\left[\ell \left(f_{h},{\tilde{Z}}_{h}\right)\right]-E_{{\bar{f}}_{h},{\bar{Z}}_{h}|Z_{h}}\left[\ell \left({\bar{f}}_{h},{\bar{Z}}_{h}\right)\right])]. \end{array}$$

By optimizing the above inequality over λ>0 and λ<0, respectively, we derive

$$\begin{array}{c} -H^{2}\sqrt{\frac{2I^{Z_{h}}\left(f_{h};{\tilde{Z}}_{h}|Z_{h}\right)}{n}}\leq E_{f_{h},{\tilde{Z}}_{h}|Z_{h}}\left[\ell \left(f_{h},{\tilde{Z}}_{h}\right)\right]-E_{{\bar{f}}_{h},{\bar{Z}}_{h}|Z_{h}}\left[\ell \left({\bar{f}}_{h},{\bar{Z}}_{h}\right)\right])]\leq H^{2}\sqrt{\frac{2I^{Z_{h}}\left(f_{h};{\tilde{Z}}_{h}|Z_{h}\right)}{n}}, \end{array}$$

and thus,

(5) $$\begin{array}{c} \left|E_{f_{h},{\tilde{Z}}_{h}|Z_{h}}\left[\ell \left(f_{h},{\tilde{Z}}_{h}\right)\right]-E_{{\bar{f}}_{h},{\bar{Z}}_{h}|Z_{h}}\left[\ell \left({\bar{f}}_{h},{\bar{Z}}_{h}\right)\right])]\right|\leq H^{2}\sqrt{\frac{2I^{Z_{h}}\left(f_{h};{\tilde{Z}}_{h}|Z_{h}\right)}{n}}. \end{array}$$

Finally, we conclude that

$$\begin{array}{cc} \left|E_{Z∼D}\left[L\left(A\left(Z_{U}\right),Z_{U}\right)-L\left(A\left(Z_{U}\right),D\right)\right]\right| & =\left|E_{Z∼D}\left[\frac{1}{H}\sum_{h=1}^{H}\ell \left(f_{h},{\tilde{Z}}_{h}\right)-E_{Z∼D}\left[\frac{1}{H}\sum_{h=1}^{H}\ell \left(f_{h},{\tilde{Z}}_{h}\right)\right]\right]\right|\\ \; & =\left|\frac{1}{H}\sum_{h=1}^{H}E_{Z_{h}∼D_{h}}\left[\ell \left(f_{h},\tilde{Z}\right)-E_{Z_{h}∼D_{h}}\left[\ell (f_{h},\tilde{Z})\right]\right]\right|\\ \; & \leq \frac{1}{H}\sum_{h=1}^{H}\left|E_{Z_{h}∼D_{h}}\left[E_{{\bar{f}}_{h},{\bar{Z}}_{h}|Z_{h}}\left[\ell \left({\bar{f}}_{h},{\bar{Z}}_{h}\right)\right])]-E_{f_{h},{\tilde{Z}}_{h}|Z_{h}}\left[\ell \left(f_{h},{\tilde{Z}}_{h}\right)\right])]\right]\right|\\ \; & \leq \frac{1}{H}\sum_{h=1}^{H}H^{2}E_{Z_{h}∼D_{h}}\left[\sqrt{\frac{2I^{Z_{h}}\left(f_{h};{\tilde{Z}}_{h}|Z_{h}\right)}{n}}\right]\;\;\;\;\;\;(\mathrm{By}\;\mathrm{Equation}\;(5))\\ \; & \leq \frac{1}{H}\sum_{h=1}^{H}H^{2}\sqrt{\frac{2E_{Z_{h}∼D_{h}}\left[I^{Z_{h}}\left(f_{h};{\tilde{Z}}_{h}|Z_{h}\right)\right]}{n}}\\ \; & =\sqrt{\frac{2H^{2}\sum_{h=1}^{H}I\left(f_{h};{\tilde{Z}}_{h}|Z_{h}\right)}{n}}\\ \; & =\sqrt{\frac{2H^{2}\sum_{h=1}^{H}I\left(f_{h};U|Z_{h}\right)}{n}}. \end{array}$$

□

Note that our setting is identical to that in [3], i.e., batch RL with value function approximation for episodic MDPs. They established a bound of the order $\tilde{O}\left(H^{2}\sqrt{\frac{1}{n}}+\sum_{h=1}^{H}R\left(F_{h}\right)\right)$, where $R(F_{h})$ represents the Rademacher complexity of the function space $F_{h}$. In contrast, our result yields an error bound of the order $O\left(H\sqrt{\frac{\sum_{h=1}^{H}I\left(f_{h};Z_{h}\right)}{n}}\right)$. As demonstrated in the subsequent section, under structural assumptions like a finite pseudo-dimension or effective dimension *d*, this bound can be refined to $\tilde{O}\left(H^{2}\sqrt{\frac{d}{n}}\right)$.

Next, we proceed to derive the high-probability version of these generalization bounds using α-mutual information.

**Theorem** **7.** *Given a dataset $Z∼D^{n}$ consists of nH samples, for any batch RL algorithm A with output $A\left(Z\right)=f=\left(f_{1},\cdots ,f_{H}\right)\in F$, if*

$$\begin{array}{c} n\geq \frac{2H^{4}}{\epsilon^{2}}\left(I_{\alpha }\left(A\left(Z\right);Z\right)+\log2+\frac{\alpha }{\alpha -1}\log\left(\frac{1}{\delta }\right)\right), \end{array}$$

*then, the generalization error for the mean squared empirical Bellman error (MSBE) loss is upper bounded by*

$$\begin{array}{c} \left|L(A(Z),Z)-L(A(Z),D)\right|\leq \epsilon \end{array}$$

*with a probability of at least 1−δ.*

**Proof.**  Let $Z=Z_{1}\cup \cdots \cup Z_{H}$ be a dataset where $Z_{h}=\left\{\left(s,a,r,s^{\prime },h\right)\right\}∼D_{h}$. Let $A\left(Z\right)=f=\left(f_{1},\cdots ,f_{H}\right)\in F$ be the output of some batch RL algorithm A. Let

$$\begin{array}{cc} L(f,Z) & =\frac{1}{H}\sum_{h=1}^{H}\ell \left(f_{h},Z_{h}\right)\\ \; & =\frac{1}{H}\sum_{h=1}^{H}\frac{1}{n}\sum_{\left(s,a,r,s^{\prime },h\right)\in Z_{h}}{\left(f_{h}\left(s,a\right)-r-V_{f_{h+1}}\left(s^{\prime }\right)\right)}^{2}. \end{array}$$

As $\ell \left(f,Z\right)\in \left[0,4H^{2}\right]$ for every *f*, it is $2H^{2}$-sub-Gaussian. By Theorem 2, we have

$$\begin{array}{c} |\ell \left(f_{h},Z_{h}\right)-E_{Z_{h}∼D_{h}}\left[\ell \left(f_{h},Z_{h}\right)\right]|\leq \epsilon \end{array}$$

with probability at least $1-\delta^{\prime }$ for

$$\begin{array}{c} n\geq \frac{8H^{4}}{\epsilon^{2}}\left(I_{\alpha }\left(f_{h};Z_{h}\right)+\log2+\frac{\alpha }{\alpha -1}\log\left(\frac{1}{\delta^{\prime }}\right)\right). \end{array}$$

As we have *n* samples at each h∈[H], we require

$$\begin{array}{c} n\geq \frac{8H^{4}}{\epsilon^{2}}\left(\max_{h}I_{\alpha }\left(f_{h};Z_{h}\right)+\log2+\frac{\alpha }{\alpha -1}\log\left(\frac{1}{\delta^{\prime }}\right)\right). \end{array}$$

The claim is now followed by the union bound by setting $\delta^{\prime }=\delta /H$. □

Recall that conditional mutual information is defined as an expectation over the KL divergence. Thus, all prior works using the CMI framework have only provided bounds on the expected generalization error. We wish to establish generalization bounds with high-probability guarantees similar to Theorem 7.

**Theorem** **8.** *Let $U\in {\left\{0,1\right\}}^{n}$ be uniformly random. Given that dataset $Z∼D^{2n}$ consists of 2nH samples, for any batch RL algorithm A with output $A\left(Z_{U}\right)=f=\left(f_{1},\cdots ,f_{H}\right)\in F$, if*

$$\begin{array}{c} n\geq \frac{8H^{4}}{\epsilon^{2}}\left(\max_{h}I_{\alpha }^{f_{h}|Z_{h}}\left(f_{h};U|Z_{h}\right)+\log2+\frac{\alpha }{\alpha -1}\log\left(\frac{H}{\delta }\right)\right). \end{array}$$

*then, the generalization error for the mean squared empirical Bellman error (MSBE) loss is upper bounded by*

$$\begin{array}{c} \left|L\left(A\left(Z_{U}\right),Z_{U}\right)-L\left(A\left(Z_{U}\right),D\right)\right|\leq \epsilon \end{array}$$

*with probability at least 1−δ.*

**Proof.**  By substituting Theorem 2 with Theorem 4 in the proof of Theorem 7, the proof is thereby obtained. □

## 5. Value Functions Under Structural Assumptions

Due to the challenges stemming from large state-action spaces, long horizons, and the temporal nature of data, there is increasing interest in identifying structural assumptions for RL with value function approximation. These works include, but are not limited to, Bellman rank [14], Witness rank [15], and Eluder dimension [16]. These structural conditions aim to develop a unified theory of generalization in RL. In this section, we demonstrate that if a function class satisfies certain structural conditions reflecting a manageable complexity, the mutual information can be effectively upper bounded.

**Definition** **11** (Covering number)**.** *The covering number of a function class $F=F_{1}\times \cdots \times F_{H}$ under metric $\rho \left(f,g\right)=\max_{h}{∥f_{h}-g_{h}∥}_{\infty }$, denoted as N(F,ϵ), is the minimum integer n, such that there exists a subset $F_{\epsilon }\subseteq F$ with $|F_{\epsilon }|=n$, and for any f∈F, there exists $g\in F_{\epsilon }$, such that ρ(x,y)≤ϵ.*

**Lemma** **2.** *For discrete random variables X,Y, and Z, we have I(X;Y|Z)≤log|X|.*

**Proof.**  Denote H(X∣Z) the conditional entropy of *X* given *Z*.

$$\begin{array}{cc} I(X;Y|Z) & =H(X|Z)-H(X|Y,Z)\\ \; & \leq H(X|Z)\;\;\;\;\;(H(X|Y,Z)\geq 0)\\ \; & =E_{z}\left[H\left(X|Z=z\right)\right]\\ \; & \leq E_{z}\left[\log|X|\right]\\ \; & =\log|X|. \end{array}$$

□

**Theorem** **9.** *Suppose the function class F has a covering number of N(F,ϵ). Let $U\in {\left\{0,1\right\}}^{n}$ be uniformly random. Given that dataset Z consists of 2nH samples, for any batch RL algorithm A with output $A\left(Z_{U}\right)=f=\left(f_{1},\cdots ,f_{H}\right)\in F$, the expected generalization error for the mean squared empirical Bellman error (MSBE) loss is upper bounded by*

$$\begin{array}{c} \left|E_{Z∼D}\left[L\left(A\left(Z_{U}\right),Z_{U}\right)-L\left(A\left(Z_{U}\right),D\right)\right]\right|\leq \sqrt{\frac{2H^{3}\log\left(|N(F,\epsilon )|\right)}{n}}+8\epsilon H+2\epsilon^{2}. \end{array}$$

**Proof.**  Let ${\tilde{Z}}_{h}={\left(Z_{h}\right)}_{U}$. We first define an oracle algorithm $A^{o}$ capable of outputting a function $A^{o}\left(Z_{U}\right)=f^{*}=\left(f_{1}^{*},\ldots ,f_{H}^{*}\right)$, such that

$$\begin{array}{c} \rho (f,f^{*})\leq \epsilon . \end{array}$$

Note that $A^{o}$ is only used for theoretical analysis. Observe that

$$\begin{array}{cc} L(A\left(Z_{U}\right),Z_{U}) & =\frac{1}{H}\sum_{h=1}^{H}\frac{1}{n}\sum_{\left(s,a,r,s^{\prime },h\right)\in {\tilde{Z}}_{h}}{\left(f_{h}\left(s,a\right)-r-V_{f_{h+1}}\left(s^{\prime }\right)\right)}^{2}\\ \; & =\frac{1}{H}\sum_{h=1}^{H}\frac{1}{n}\sum_{\left(s,a,r,s^{\prime },h\right)\in {\tilde{Z}}_{h}}{\left(f_{h}\left(s,a\right)-f_{h}^{*}\left(s,a\right)+f_{h}^{*}\left(s,a\right)-r-V_{f_{h+1}}\left(s^{\prime }\right)\right)}^{2}\\ \; & =\epsilon^{2}+\frac{1}{H}\sum_{h=1}^{H}\frac{1}{n}\sum_{\left(s,a,r,s^{\prime },h\right)\in {\tilde{Z}}_{h}}{\left(f_{h}^{*}\left(s,a\right)-r-V_{f_{h+1}}\left(s^{\prime }\right)\right)}^{2}\\ \; & \;+2\epsilon \frac{1}{H}\sum_{h=1}^{H}\frac{1}{n}\sum_{\left(s,a,r,s^{\prime },h\right)\in {\tilde{Z}}_{h}}\left(f_{h}^{*}\left(s,a\right)-r-V_{f_{h+1}}\left(s^{\prime }\right)\right)\\ \; & \leq \epsilon^{2}+L\left(A^{o}\left(Z_{U}\right),Z_{U}\right)+4\epsilon H. \end{array}$$

Thus,

$$\begin{array}{c} L\left(A\left(Z_{U}\right),Z_{U}\right)-L\left(A^{o}\left(Z_{U}\right),Z_{U}\right)\leq 4\epsilon H+\epsilon^{2}. \end{array}$$

Bounding $|L\left(A\left(Z_{U}\right),D\right)-L\left(A^{o}\left(Z_{U}\right),D\right)|$ is similar. Now, we have

$$\begin{array}{cc} L\left(A\left(Z_{U}\right),Z_{U}\right)-L\left(A\left(Z_{U}\right),D\right) & =L\left(A\left(Z_{U}\right),Z_{U}\right)-L\left(A^{o}\left(Z_{U}\right),Z_{U}\right)+L\left(A^{o}\left(Z_{U}\right),Z_{U}\right)\\ \; & \;-L\left(A^{o}\left(Z_{U}\right),D\right)+L\left(A^{o}\left(Z_{U}\right),D\right)-L\left(A\left(Z_{U}\right),D\right). \end{array}$$

As $|L\left(A\left(Z_{U}\right),Z_{U}\right)-L\left(A^{o}\left(Z_{U}\right),Z_{U}\right)|\leq \epsilon$ and $|L\left(A\left(Z_{U}\right),D\right)-L\left(A^{o}\left(Z_{U}\right),D\right)|\leq \epsilon$, we have

$$\begin{array}{c} L\left(A\left(Z_{U}\right),Z_{U}\right)-L\left(A\left(Z_{U}\right),D\right)\leq L\left(A^{o}\left(Z_{U}\right),Z_{U}\right)-L\left(A^{o}\left(Z_{U}\right),D\right)+8\epsilon H+2\epsilon^{2}. \end{array}$$

By Theorem 6,

$$\begin{array}{cc} \left|E_{Z∼D}\left[L\left(A^{o}\left(Z_{U}\right),Z_{U}\right)-L\left(A^{o}\left(Z_{U}\right),D\right)\right]\right| & \leq \sqrt{\frac{2H^{2}\sum_{h=1}^{H}I\left(f_{h}^{*};U|Z_{h}\right)}{n}}\\ \; & \leq \sqrt{\frac{2H^{2}\sum_{h=1}^{H}\log(|F_{\epsilon }\left|\right)}{n}}\;\;\;(\mathrm{By}\;\mathrm{Lemma}\;2)\\ \; & =\sqrt{\frac{2H^{3}\log(|F_{\epsilon }\left|\right)}{n}}\\ \; & =\sqrt{\frac{2H^{3}\log\left(|N(F,\epsilon )|\right)}{n}}. \end{array}$$

Therefore,

$$\begin{array}{c} \left|E_{Z∼D}\left[L\left(A\left(Z_{U}\right),Z_{U}\right)-L\left(A\left(Z_{U}\right),D\right)\right]\right|\leq \sqrt{\frac{2H^{3}\log\left(|N(F,\epsilon )|\right)}{n}}+8\epsilon H+2\epsilon^{2}. \end{array}$$

□

Structural assumptions on the function space typically entail a finite covering number. Next, we consider the simplest case: the pseudo-dimension. The pseudo-dimension is a complexity measure of real-valued function classes, analogous to the VC dimension used for binary classification. Although the value function space may be infinite, it remains learnable if it has a finite pseudo-dimension.

**Definition** **12** (VC-Dimension [17])**.** *Given hypothesis class H⊆X→{0,1}, its VC-dimension VCdim(H) is defined as the maximal cardinality of a set $X=\{x_{1},\ldots ,x_{\left|X\right|}\}\subseteq X$ that satisfies $|H_{X}|=2^{\left|X\right|}$ (or X is shattered by H), where $H_{X}$ is the restriction of H to X, namely $\left\{\left(h\left(x_{1}\right),\ldots ,h\left(x_{\left|X\right|}\right)\right):h\in H\right\}$.*

**Definition** **13** (Pseudo dimension [18])**.** *Suppose X is a feature space. Given hypothesis class H⊆X→R, its pseudo dimension Pdim(H) is defined as $\mathrm{Pdim}\left(H\right)=\mathrm{VCdim}\left(H^{+}\right)$, where $H^{+}=\left\{\left(x,\xi \right)\mapsto 1\left[h\left(x\right)>\xi \right]:h\in H\right\}\subseteq X\times R\to \left\{0,1\right\}\}$.*

**Lemma** **3** (Bounding covering number by pseudo dimension [19])**.** *Given hypothesis class H⊆X→R with Pdim(H)≤d, we have*

logN(H,ϵ)≤O(dlog(1/ϵ)).

**Corollary** **1.** *Suppose the function class $F_{h}\subset F$ has a finite pseudo dimension $\mathrm{Pdim}(F_{h})=d$. For any batch RL algorithm with n training samples, the expected generalization error for the mean squared empirical Bellman error (MSBE) loss is upper bounded by $\tilde{O}\left(H^{2}\sqrt{d/n}\right)$.*

**Proof.**  As $\mathrm{Pdim}(F_{h})=d$ and $F=F_{1}\times \cdots \times F_{H}$, we have logN(F,ϵ)≤O(dHlog(1/ϵ)). The claim follows from Theorem 9 by setting $\epsilon =H\sqrt{\frac{d}{n}}$. □

A prior study on finite sample guarantees for minimizing the Bellman error, using pseudo-dimension, demonstrated a sample complexity with a dependence of $\tilde{O}\left(d^{2}\right)$ [5]. In contrast, our sample complexity exhibits a dependence of $\tilde{O}\left(d\right)$ on the pseudo-dimension.

Now, we introduce another complexity measure known as the effective dimension [20], which has a similar covering number to the pseudo-dimension. The effective dimension quantifies how the function class responds to data, indicating the minimum number of samples required to learn effectively.

**Definition** **14** (ϵ-effective dimension of a set [20])**.** *The ϵ-effective dimension of a set X is the minimum integer $d_{\mathrm{eff}}\left(X,\epsilon \right)=n$, such that*

$$\underset{x_{1},\ldots ,x_{n}\in X}{\sup}\frac{1}{n}\log\det\left(I+\frac{1}{\epsilon^{2}}\sum_{i=1}^{n}x_{i}x_{i}^{⊤}\right)\leq e^{-1}.$$

**Definition** **15** (ϵ-effective dimension of a function class [20])**.** *Given a function class F defined on X, its ϵ-effective dimension $d_{\mathrm{eff}}\left(F,\epsilon \right)=n$ is the minimum integer n, such that there exists a separable Hilbert space H and a mapping ϕ:X→H, so that*

- *for every f∈F, there exists $\theta_{f}\in B_{H}\left(1\right)$ satisfying $f\left(x\right)={\langle \theta_{f},\phi \left(x\right)\rangle }_{H}$ for all x∈X,*
- *$d_{\mathrm{eff}}\left(\phi \left(X\right),\epsilon \right)=n$, where ϕ(X)={ϕ(x):x∈X}.*

**Definition** **16** (Kernel MDPs [21])**.** *In a kernel MDP of effective dimension d, for each step h∈[H], there exist feature mappings $\phi_{h}:S\times A\to H$ and $\psi_{h}:S\to H$, where H is a separable Hilbert space, so that the transition measure can be represented as the inner product of features, i.e.,*

$$P_{h}\left(s^{\prime }|s,a\right)={\langle \phi_{h}\left(s,a\right),\psi_{h}\left(s^{\prime }\right)\rangle }_{H}.$$

*Besides, the reward function is linear in ϕ, i.e.,*

$$r_{h}\left(s,a\right)={\langle \phi_{h}\left(s,a\right),\theta_{h}^{r}\rangle }_{H}$$

*for some $\theta_{h}^{r}\in H$. Here, ϕ is known to the learner while ψ and $\theta_{h}^{r}$ are unknown. Moreover, a kernel MDP satisfies the following regularization conditions: for all h*

- *$∥\theta_{h}^{r}{∥}_{H}\leq 1$ and $∥\phi_{h}{\left(s,a\right)∥}_{H}\leq 1$ for all s,a.*
- *${∥\sum_{s\in S}V\left(s\right)\psi_{h}\left(s\right)∥}_{H}\leq 1$ for any function V:S→[0,1].*
- *$\dim_{\mathrm{eff}}\left(X_{h},\epsilon \right)\leq d$ for all h, where $X_{h}=\left\{\phi_{h}\left(s,a\right):\left(s,a\right)\in S\times A\right\}$.*

Kernel MDPs are extensions of the traditional MDPs where the transition dynamics and rewards are represented in a Reproducing Kernel Hilbert Space (RKHS). In this setup, the value functions or Q-functions are approximated using kernel methods, allowing the model to capture more complex dependencies in the data compared to linear models. To learn kernel MDPs, it is necessary to construct a function class F.

**Lemma** **4** (Bounding covering number by effective dimension [21])**.** *Let M be a kernel MDP of effective dimension d, then*

logN(F,ϵ)≤O(Hdlog(1+dH/ϵ)).

**Corollary** **2.** *Suppose the function class F has a finite effective dimension d. For any batch RL algorithm with n training samples, the expected generalization error for the mean squared empirical Bellman error (MSBE) loss is upper bounded by $\tilde{O}\left(H^{2}\sqrt{d/n}\right)$.*

We showed that when a function class contains infinitely many elements, a finite covering number can be used to upper bound the generalization error. Just as the VC-dimension imposes a finite cardinality, various concepts in real-valued function classes, such as pseudo-dimension and effective dimension, result in a finite covering number, thereby ensuring efficient learning.

## 6. Discussion

In this paper, we analyzed the generalization property of batch reinforcement learning within the framework of information theory. We established generalization bounds using both conditional and unconditional mutual information. Besides, we demonstrated how to leverage the structure of the function space to guarantee generalization. Due to the merits of the information-theoretic approach, there are several appealing future research directions.

The first interesting avenue is to extend the results to the online setting. It is noteworthy that in on-policy learning, the inputs (e.g., the reward and the next state), are influenced by the output (e.g., the policy or the model), which highlights a significant disparity compared to off-policy and supervised learning. In supervised learning, a small mutual information between the input and the output indicates that the model is not overfitting. In on-policy learning, analyzing the mutual information between the input and the output can be more complicated and insightful. For example, in model-based reinforcement learning, where the model is a part of the output, a small mutual information might indicate that the learned model focuses more on the goal of maximizing the cumulative reward rather than solely capturing the transition dynamics. How to learn an effective model beyond merely fitting the transition is the central theme in decision-aware model-based reinforcement learning [22,23,24,25,26,27,28].

As in the supervised learning setting, where various algorithms such as Stochastic Gradient Descent (SGD) [29] and Stochastic Gradient Langevin Dynamics (SGLD) have been studied [30], a promising future direction is to analyze information-theoretic generalization bounds for specific reinforcement learning algorithms such as stochastic policy gradient methods.

In addition, the information-theoretic approach has the potential to unify various concepts related to generalization, such as differential privacy and stability [12,31]. It would be interesting to explore how these notions in reinforcement learning can be leveraged to guarantee generalization.

Analyzing generalization for reinforcement learning is inherently more challenging than in supervised learning [32,33,34]. Therefore, we hope that the information-theoretic approach will provide more insights into understanding the generalization of reinforcement learning.

## Data Availability

No data were created or analyzed in this theoretical study. Data sharing is not applicable.

## Appendix A. Related Work

### Appendix A.1. Batch Reinforcement Learning

A body of literature focuses on finite sample guarantees for batch reinforcement learning with function approximation [35,36,37,38,39,40]. Common assumptions in batch RL, such as concentrability, realizability, and completeness, have also been examined in more recent studies [41,42,43]. The most relevant work to ours [3] investigates the generalization performance of batch RL under the same setting using Rademacher complexities.

### Appendix A.2. Structural Conditions for Efficient RL

Analogous to complexity measures in supervised learning, several structural conditions have been studied to enable efficient reinforcement learning, including Bellman rank [14], Witness rank [15], Eluder dimension [16], Bellman Eluder dimension [21], and more [20,37,44]. Identifying structural conditions and classifying RL problems clarifies the limits of what can be learned and guides the design of efficient algorithms.

### Appendix A.3. Information-Theoretic Study of Generalization

The information-theoretic approach was initially introduced by [1,2] and subsequently refined to derive tighter bounds [45,46,47]. Besides, various other information-theoretic bounds have been proposed, leveraging concepts such as conditional mutual information [12], *f*-divergence [11], the Wasserstein distance [48,49], and more [50,51]. Some studies have focused on analyzing specific algorithms [29,30,52,53,54,55] while others have examined particular settings such as deep learning [56], iterative semi-supervised learning [57], transfer learning [58], and meta-learning [59,60]. There are also works attempting to provide a unified framework for generalization from an information-theoretic perspective [31,61,62].
